# Towards Optimal Cardiovascular Health: A Comprehensive Review of Preventive Strategies

**DOI:** 10.7759/cureus.60877

**Published:** 2024-05-22

**Authors:** Philip Branigan, Y. V Duong, Ammar Y Abdulfattah, Jacob Sabu, Mary Mallappallil, Sabu John

**Affiliations:** 1 Department of Cardiology, State University of New York Downstate Medical Center, Brooklyn, USA; 2 Department of Cardiology, University of Debrecen Medical School, Debrecen, HUN; 3 Department of Internal Medicine, State University of New York Downstate Medical Center, Brooklyn, USA; 4 Department of Cardiology, State University of New York Downstate Health Sciences University, Brooklyn, USA; 5 Department of Nephrology, State University of New York Downstate Medical Center, Brooklyn, USA

**Keywords:** diabetes, cholesterol, exercise, diet, hypertension, heart disease prevention

## Abstract

Heart disease remains a prominent global health concern, with cardiovascular disease (CVD) standing as a leading cause of death worldwide. Preventing heart disease not only decreases the risk of premature death but also mitigates complications like heart attacks, strokes, and arrhythmias, thereby enhancing overall health and quality of life. The economic burden of heart disease treatment highlights the importance of implementing preventive measures, such as lifestyle changes and early interventions, which can alleviate healthcare costs. These strategies, targeting risk factors like hypertension (HTN), diabetes mellitus (DM), dyslipidemia, and obesity, not only prevent heart disease but also reduce the risk of other health issues. Herein, this review covers various preventive measures, including dietary interventions, exercise, controlling HTN, DM, cholesterol, and weight, smoking cessation, and pharmacological interventions. By critically analyzing the guidelines and leveraging robust data alongside variations in recommendations, this review aims to elucidate effective primary prevention strategies for CVD.

## Introduction and background

Heart disease stands as one of the leading causes of death worldwide. Prevalent cases of total cardiovascular disease (CVD) nearly doubled from 271 million in 1990 to 523 million in 2019 [1]. Moreover, the number of CVD-related deaths steadily increased, from 12.1 million in 1990 to 18.6 million in 2019 [1]. Heart disease can lead to various complications, including heart attacks, strokes, heart failure, and arrhythmias. Preventing heart disease mitigates the risk of these complications, thereby enhancing quality of life and improving overall health [2].

Treatment for heart disease and its associated complications results in substantial healthcare costs [3]. Implementing preventive measures, such as lifestyle modifications and early interventions, has the potential to alleviate the economic burden on healthcare systems and individuals [4]. Heart disease can significantly impede an individual's daily life, limiting physical abilities and causing discomfort [5]. Prevention measures empower individuals to maintain an active, healthy lifestyle, thereby facilitating improved physical and mental well-being. These strategies primarily target managing risk factors like high cholesterol, high blood pressure, diabetes, obesity, and smoking [6]. Educating individuals about heart disease risk factors, symptoms, and preventive measures encourages them to take charge of their health, promoting early detection and effective intervention [7].

## Review

The 2019 American College of Cardiology and American Heart Association (ACC/AHA) guideline on primary prevention of CVD aims to offer evidence-based recommendations for preventing CVD in individuals without a history of the condition. This guideline primarily emphasizes lifestyle factors, other determinants affecting CVD risk, patient-centered approaches, and considerations regarding the cost and value of primary prevention [8]. Its goal is to ensure optimal dissemination of information and aid clinicians and individuals in making informed decisions to prevent CVD [8].

The ACC/AHA 2019 guideline serves as a resource for healthcare professionals seeking to prevent CVD in patients. Within this guideline, several significant recommendations are highlighted, including the promotion of a collaborative approach to care involving teams in managing risk factors associated with atherosclerotic CVD (ASCVD). Additionally, there is an emphasis on supporting shared decision-making during discussions concerning the most suitable strategies for lowering ASCVD risk. Moreover, the guideline stresses the importance of integrating social determinants of health into treatment plans to enhance recommendations for preventing ASCVD [8].

These recommendations underscore the significance of adopting a healthy lifestyle, comprising dietary habits, physical activity, and addressing other factors influencing CVD risk like obesity, diabetes mellitus (DM), blood cholesterol, high blood pressure, smoking, and aspirin use [9]. The guideline emphasizes patient-centered care, integrating team-based care delivery, shared decision-making, and recognizing social determinants of health in ASCVD prevention efforts [8]. The social determinants of health are “the structural determinants and conditions in which people are born, grow, live, work, and age” [9]. These structural determinants and conditions include the physical environment, socioeconomic status, education, employment, and social support networks, as well as access to healthy food and health care.

Healthcare professionals can effectively implement the recommendations outlined in the 2019 ACC/AHA guidelines through several strategies. Adopting a team-based care approach involves leveraging multidisciplinary health professionals to enhance the quality and sustainability of ASCVD prevention [8]. It facilitates collaboration among various clinicians, promotes clinical decision-making, and encourages patient and family engagement to support treatment goals [8].

Another strategy involves integrating shared decision-making into patient discussions regarding optimal ASCVD risk reduction strategies. This approach actively engages patients in the decision-making process, considering their values, preferences, and associated health conditions [8]. Additionally, healthcare professionals should account for social determinants of health while implementing treatment recommendations for ASCVD prevention [8]. Understanding the social and economic factors influencing patients' health can tailor interventions and enhance outcomes [8].

Fegers-Wustrow et al. conducted a comparative analysis between the 2019 ACC/AHA guidelines and the 2021 European Society of Cardiology (ESC) guidelines. Both the ESC and ACC/AHA guidelines acknowledge the significance of addressing nonclassical risk factors for CVD. While the ESC guideline adopts a holistic approach, extending risk assessment beyond individual patients to encompass global population-level risk, it recognizes the impact of environmental factors like air and noise pollution, workplace environment, and city planning on CVD risk [10]. The ESC guideline advocates legislative action at various governance levels to address these nonclassical risk factors [10].

Diet and cardiovascular health

Embracing healthy eating patterns such as the Mediterranean, Dietary Approaches to Stop Hypertension (DASH), or plant-based diets can notably decrease the likelihood of CVD. These dietary approaches, including the Mediterranean diet, which advocates for a rich intake of fruits, vegetables, whole grains, nuts, seeds, and healthy fats like olive oil, with moderate consumption of fish and poultry while limiting red meat and processed foods [11]; the DASH diet, which prioritizes fruits, vegetables, whole grains, lean proteins, and low-fat dairy to lower blood pressure and reduce heart disease risk [12,13]; and the plant-based diet, which mainly consists of plant-derived foods such as vegetables, fruits, legumes, nuts, and seeds, with minimal or no animal products, have been associated with a reduced risk of CVD [14]. Focusing on the quality of nutrients is crucial, prioritizing foods rich in fiber, antioxidants, vitamins, and minerals while minimizing saturated fats, trans-fats, sodium, and added sugars [14]. Managing cholesterol and fat intake involves limiting saturated and trans fats to lower low-density lipoprotein cholesterol (LDL-C) levels while advocating for healthy fats like those found in olive oil, avocados, nuts, and seeds [11]. To maintain healthy blood pressure, reducing sodium intake by avoiding processed and high-sodium foods is essential [14]. Moderation in alcohol consumption is emphasized, as excessive intake can elevate blood pressure and contribute to heart disease [15]. Portion control and balanced meals are encouraged to manage weight, considering obesity as a significant risk factor for heart disease [15]. Adopting a heart-healthy diet can lower the risk of developing CVD, help manage existing conditions like high blood pressure and cholesterol, and contribute to overall well-being [15]. It is essential to consult with a healthcare professional or a registered dietitian for personalized dietary recommendations based on individual health needs and goals [15].

Mediterranean Diet

The Mediterranean diet's richness in monounsaturated fatty acids, predominantly from sources like olive oil, contributes to diminishing LDL-C levels and ameliorating overall lipid profiles [11]. Abundant consumption of fruits, vegetables, and nuts within this dietary pattern furnishes a broad spectrum of antioxidants and anti-inflammatory agents, thus mitigating inflammation and oxidative stress within the body [11]. Moreover, this diet's association with enhanced insulin sensitivity facilitates the regulation of blood sugar levels, reducing the susceptibility to metabolic conditions such as type 2 DM [11]. By amalgamating healthy fats, fiber, and antioxidants, the Mediterranean diet contributes to cardiovascular well-being by thwarting blood clot formation, diminishing blood pressure, and curbing the likelihood of CVDs [11]. Enriched with phytochemicals, vitamins, and minerals renowned for their anti-cancer attributes, this dietary regimen potentially lowers cancer risks [11].

Various studies underscore the Mediterranean diet's correlation with reduced blood pressure and improved cholesterol profiles, highlighting its composition abundant in fruits, vegetables, whole grains, legumes, and healthy fats like olive oil [11-15]. Emphasizing lean protein sources like fish and poultry while limiting red meat and processed food intake, coupled with reduced consumption of saturated and trans-fats commonly found in fried and processed foods, aids in cholesterol level management [15]. Incorporating foods high in soluble fiber, like oats, barley, and legumes, also improves cholesterol levels [15]. 

The concept of synergistic effects of various foods and nutrients on cardiovascular health postulates that certain food and nutrient combinations exert a more substantial positive impact when consumed together than when individually consumed [16]. This concept highlights the holistic quality of dietary patterns over singular nutrients or foods, accentuating their collective potential in promoting cardiovascular health [16].

Jimenez-Torres et al. delve into two preventive strategies against coronary artery disease (CAD): the Mediterranean diet and a low-fat diet [17]. The Mediterranean dietary pattern has exhibited numerous health advantages, notably a reduced risk of CVD. In the context of the study, adhering to a Mediterranean diet demonstrated improvements in endothelial function and bolstered endothelial repair mechanisms [17]. This finding suggests the Mediterranean diet’s potential to safeguard the endothelium and decrease cardiovascular event risks in individuals with CAD. The low-fat diet advocated in the study aligns with the AHA guidelines, focusing on decreasing total fat, cholesterol, and saturated fats while augmenting complex carbohydrate intake [8-17]. However, in contrast to the Mediterranean diet, the low-fat diet did not exhibit comparable positive effects on endothelial function [17]. The study found the Mediterranean diet to be more efficacious in improving endothelial function and fostering a superior balance in endothelial vascular homeostasis [17]. Overall, the findings propose that adopting a Mediterranean diet might constitute the optimal preventive strategy against CAD, primarily due to its efficacy in enhancing endothelial function and diminishing cardiovascular event risks in individuals with CAD [17].

DASH Diet

The DASH diet is a specialized regimen designed to mitigate elevated blood pressure and enhance cardiovascular well-being [12,13]. It advocates heightened consumption of fruits and vegetables rich in potassium, magnesium, and fiber [12,13]. These nutrients play a pivotal role in blood pressure regulation and diminishing CVD risks [12,13]. Essential components of whole grains, like brown rice, whole wheat bread, and oats, integral to the DASH diet, provide ample fiber and heart-supporting nutrients, contributing to reduced cholesterol levels. Encouraging the intake of lean proteins such as poultry, fish, beans, and nuts [12,13], which are lower in saturated fats compared to red and processed meats, assists in managing cholesterol levels. Additionally, the DASH diet recommends low-fat or fat-free dairy products that are rich in calcium and protein while maintaining a lower saturated fat content compared to full-fat dairy options [12,13].

The DASH diet advocates limiting sodium intake, which is crucial for blood pressure management, preventing fluid retention, and reducing cardiovascular strain [12,13]. Studies affirm the DASH diet's efficacy in reducing blood pressure, improving cholesterol profiles, and decreasing CVD risks [18]. Its emphasis on whole, nutrient-rich foods and sodium restriction renders it an efficacious dietary regimen, particularly for individuals managing or preventing hypertension (HTN) [18]. Notably, among participants initially averaging a systolic blood pressure of 130 mm Hg, the DASH-type diets elicited a 10 mm Hg reduction in blood pressure [18]. Nonetheless, the effectiveness of the DASH diet may fluctuate based on individual adherence and baseline blood pressure levels [18].

While sharing commonalities, the DASH diet and a fruit- and vegetable-focused diet (F/V) exhibit distinctive features. The former encompasses fruits, vegetables, whole grains, lean proteins, and low-fat dairy, targeting blood pressure reduction and CVD prevention through controlled sodium, saturated fat, and sugar intake [19]. Conversely, the F/V diet emphasizes a diverse array of nutrient-dense plant-based foods, advocating a spectrum of colors and varieties in fruit and vegetable consumption [19].

An investigation on their impact on 10-year ASCVD risk scores observed that both the DASH and F/V diets reduced risks by approximately 10% over eight weeks compared to a typical American diet [19]. Notably, the DASH diet exhibited a more pronounced reduction in systolic blood pressure, while the F/V diet did not significantly affect blood pressure levels. Furthermore, the DASH diet resulted in decreased high-density lipoprotein cholesterol (HDL-C) levels, whereas the F/V diet did not influence HDL-C [19].

Vegetarian Diet (Plant-Based)

A vegetarian diet, particularly when carefully structured and centered on whole, nutrient-dense foods, holds the potential to yield several advantages for cardiovascular well-being [14]. Typically, well-organized vegetarian diets contain lower levels of saturated fat and cholesterol in comparison to meat-inclusive diets [14]. This distinction may contribute to reduced LDL-C ("bad") cholesterol levels, thereby mitigating the risk associated with arterial plaque buildup [14]. Emphasizing fruits, vegetables, whole grains, legumes, nuts, and seeds, vegetarian diets are rich in fiber, antioxidants, vitamins, and minerals, facilitating blood pressure reduction, enhancing blood vessel function, and attenuating inflammation, supporting overall heart health [14]. Often accentuating healthy fats present in avocados, nuts, seeds, and olive oil [14], particularly monounsaturated and polyunsaturated fats, these diets might ameliorate cholesterol profiles and diminish the susceptibility to heart disease. Additionally, their association with reduced body weight and a lowered risk of obesity contributes to enhanced heart health, reducing the likelihood of conditions like high blood pressure and type 2 DM [14].

A systematic review and meta-analysis conducted by the European Journal of Nutrition, encompassing 13 prospective cohort studies, explored the correlation between vegetarian diets and CVD, ischemic heart disease, and stroke [20]. The findings highlighted a lower risk of CVD and ischemic heart disease associated with vegetarian diets in comparison to non-vegetarian diets. However, no significant association was observed between vegetarian diets and stroke risk [20], suggesting potential protective effects against certain cardiovascular ailments. Nevertheless, additional research is imperative to comprehensively comprehend the relationship between vegetarian diets and stroke risk [20].

Contrasting evidence emerges from another systematic review regarding the cardiovascular advantages of a vegan diet [21]. While certain studies suggest potential benefits, such as averting recurrent cardiovascular events and exhibiting reduced carotid artery intima-media thickness, others fail to establish a substantial connection between adherence to a vegan diet and the risks associated with primary cardiovascular events, or CAD [22]. Interestingly, one study hints at a potentially elevated risk of ischemic stroke among vegans compared to those consuming animal products [23]. Overall, the limited number of high-quality studies has weakened the evidence supporting the role of a vegan diet in CVD prevention or development [21].

Obesity and CVD

Obesity, defined as excessive fat accumulation in adipose tissue, poses significant health risks [24]. It is a well-established risk factor for CVD [24]. Waist circumference serves as an optimal indicator of visceral fat and is superior in predicting metabolic syndromes like DM, HTN, and dyslipidemia [25]. Inflammatory cytokines such as tumor necrosis factor-alpha (TNF-𝛼), CRP, and interleukins (IL-6, IL-18), along with resistin and visfatin, are dysregulated in obesity [26,27]. Elevated levels of these markers are associated with increased CVD risk, potentially contributing to its pathogenesis [28-30]. These changes are linked to insulin resistance, HTN, atherosclerosis, CVD, and certain cancers [31,32].

Visceral adipose tissue and pericardial fat are implicated in CAD and myocardial infarction, likely through systemic and vascular inflammation promoting atherosclerosis [33,34]. Obese adolescents with elevated inflammatory markers and low adiponectin levels face increased risks of CAD and type 2 DM [35]. Studies such as the Cholesterol and Recurrent Events Trial have shown that elevated CRP levels post-myocardial infarction are linked to coronary events [36]. Dietary interventions and exercise for weight loss, as reviewed here, can effectively reduce inflammation and decrease CVD incidence [37].

Exercise and cardiovascular health

Physical activity stands as a fundamental element of cardiovascular well-being, yielding a plethora of advantages that positively influence both the heart and blood vessels [38]. Consistent exercise strengthens the cardiac muscle, enhancing its efficiency in pumping blood across the body, therefore reducing the resting heart rate and alleviating strain on the heart [38]. Its role in managing blood pressure is evident through the enhancement of blood vessel flexibility and health, mitigating resistance to blood flow, and resulting in reduced overall blood pressure levels [38]. Additionally, physical activity aids in averting arterial plaque buildup by improving cholesterol levels and curbing inflammation, subsequently diminishing the risk of health disease associated with atherosclerosis [39,40].

According to the AHA guidelines, it is recommended to engage in at least 150 minutes of moderate-intensity aerobic exercise or 75 minutes of vigorous-intensity aerobic exercise weekly, coupled with muscle-strengthening activities on two or more days [40]. Activities encompassing brisk walking, running, cycling, swimming, and strength training all contribute substantially to improved cardiovascular health [41]. Before initiating a new exercise regimen, particularly for individuals with pre-existing health conditions, seeking counsel from a healthcare provider is advisable to ensure safety and receive personalized exercise guidance [42]. Interestingly, a study that looked into the relative effects of concentrated exercise in one to two days per week, also referred to as Weekend Warriors, versus more evenly distributed activity over four to five days or the whole week concluded that concentrated exercise within one to two days was associated with a similarly lower risk of cardiovascular outcomes to more evenly distributed activity [43].

When coupled with a balanced diet, exercise contributes significantly to weight control or weight reduction [44]. The positive effect of physical activity extends to enhanced blood circulation, ensuring efficient delivery of oxygen and nutrients to the tissues and organs of the body, including the heart [38-45].

Preventive Effects of Exercise on CAD

Exercise assumes a key role in the prevention of CAD, offering a multitude of preventive advantages. Consistent physical activity enhances cardiovascular fitness by fortifying the heart muscle and augmenting its efficiency in pumping blood [38]. This improvement diminishes the cardiac workload and mitigates the risk of CAD development. In addition, exercise effectively manages various CAD-related risk factors, including elevated cholesterol levels, high blood pressure, obesity, and DM [38-45], thereby contributing to a reduced chance of CAD onset [38].

Empirical evidence consistently supports the protective impacts of exercise against CAD [42]. A blend of aerobic exercises such as walking, jogging, cycling, or swimming, coupled with strength training, promotes significant benefits in CAD prevention and management [42]. Moreover, exercise exhibits the potential to slow or avert atherosclerosis progression, characterized by arterial plaque buildup [38-45]. Regular physical activity helps in improving cholesterol profiles, dampening inflammation, and thwarting arterial rigidity, collectively reducing the risk of heart disease associated with atherosclerosis [38]. Enhancing blood circulation through exercise facilitates enhanced oxygen delivery to the heart muscle, thereby diminishing the chance of ischemic heart disease, which stems from insufficient blood and oxygen supply due to narrowed arteries [38-45].

Furthermore, consistent engagement in exercise plays a crucial role in regulating heart rate and blood pressure, fostering healthy levels of these crucial indicators. This not only alleviates strain on the heart and blood vessels but also provides reassurance about lowering the risk of CAD [46].

Strategies to reduce the risk of sudden cardiac death during exercise

Sudden cardiac death (SCD) signifies an unforeseen and sudden cessation of heart function, typically arising from an electrical disturbance that halts its beating [47]. This event occurs within minutes of symptom onset and frequently results in mortality if not promptly addressed [47].

Individuals engaged in habitual exercise often exhibit heightened body awareness, enabling them to discern potential warning signs of cardiac issues [38]. This perceptiveness prompts timely medical intervention, potentially averting instances of SCD [38]. Nonetheless, in specific scenarios, vigorous or intense physical activity, particularly among individuals with pre-existing heart conditions, may trigger SCD [48]. While this risk remains relatively low within the general populace, it escalates in individuals harboring underlying cardiac conditions [48].

Before initiating an exercise regimen, individuals with established cardiac conditions or predisposing factors should engage in consultations with healthcare providers to evaluate whether specific exercise types and intensities are appropriate for them [42]. Incrementally elevating exercise duration and intensity over time helps the body acclimatize and diminishes the probability of sudden cardiac strain [38]. Recognizing indicators like chest discomfort, lightheadedness, breathlessness, or irregular heartbeats during physical activity is crucial, warranting immediate medical attention when noticed [42].

Implementation of proper warm-up and cool-down routines preceding and following exercise sessions serves to prime the heart and alleviate stress on the cardiovascular system [49]. Although exercise yields multifaceted advantages for cardiac well-being, individuals with established cardiac ailments or heightened susceptibility to SCD should seek advice from healthcare professionals to ascertain a safe exercise regimen that is tailored to their needs [49]. This personalized strategy aids in curtailing the risk of SCD during physical exertion [49].

Congestive heart failure: risk factors and prevention

Congestive heart failure (CHF) occurs when the heart muscle becomes weakened and cannot pump blood as effectively as it should [50]. Various risk factors contribute to the development of CHF. CAD, characterized by blockages in the coronary arteries leading to heart attacks, poses a significant risk as it can damage the heart muscle, heightening the likelihood of CHF. HTN is another prominent risk factor, placing increased strain on the heart over time, potentially leading to CHF. Previous heart attacks also elevate the risk by causing damage to the heart muscle. Malfunctioning heart valves can force the heart to work harder, increasing the risk of CHF, while conditions like cardiomyopathy directly affecting the heart muscle can also contribute to CHF [50,51]. Additionally, uncontrolled DM can harm blood vessels and nerves, impairing the heart's function and increasing the risk of CHF. Obesity, through its association with conditions like HTN and DM, further strains the heart, contributing to the risk of CHF [50].

Addressing HTN through lifestyle modifications and medication significantly reduces the risks associated with CHF [51]. Managing blood sugar levels via dietary adjustments, exercise, medications, and consistent monitoring plays an important role in preventing DM-related complications associated with CHF [52].

Regular health assessments aid in monitoring conditions such as CAD, HTN, and other potential risk factors for CHF [53]. Early identification and management of risk factors, combined with lifestyle adjustments and appropriate medical intervention, substantially delay or prevent the onset of CHF [6]. Consistent consultations with healthcare professionals for personalized guidance and monitoring are fundamental aspects of strategies aimed at preventing CHF [54]. Adhering to prescribed medications under healthcare supervision is essential to prevent further cardiac damage that could lead to CHF [54].

Relationship between HTN and CAD

Both HTN and CAD share common risk factors, including obesity, DM, high cholesterol levels, smoking, and an unhealthy lifestyle [46]. These factors contribute to the development and progression of both HTN and CAD [46]. HTN causes damage to the walls of blood vessels, making them less flexible and more susceptible to atherosclerosis (narrowing due to plaque buildup) [55]. This atherosclerosis can affect the coronary arteries, leading to CAD [55].

HTN accelerates the process of atherosclerosis, which can lead to the buildup of plaque in the coronary arteries [55]. These blockages restrict blood flow to the heart, causing angina (chest pain) or leading to a heart attack [55]. HTN is a significant risk factor for heart attacks, heart failure, and other complications associated with CAD [56]. Uncontrolled HTN significantly raises the risk of adverse cardiovascular events. Controlling HTN is essential to managing CAD and preventing its progression [57]. Addressing high blood pressure through lifestyle changes and medications can slow down the development of CAD and reduce the risk of associated complications [58].

Managing both HTN and CAD involves lifestyle modifications, medications (if prescribed), regular medical check-ups, and adherence to treatment plans [59]. Consulting with healthcare providers for personalized guidance and treatment strategies tailored to individual health needs is essential in managing this relationship between HTN and CAD [60].

Prevention Strategies for HTN

Regularly checking your blood pressure can help detect any changes or abnormalities early on. Following a balanced diet that is low in sodium, saturated fats, and cholesterol can help prevent HTN [61]. Engaging in regular physical activity, such as brisk walking, jogging, swimming, or cycling, can help lower blood pressure and maintain a healthy weight [61]. Maintaining a healthy weight or losing weight if overweight can significantly reduce the risk of developing HTN. Additionally, excessive alcohol consumption can raise blood pressure [61].

Smoking can raise blood pressure and damage blood vessels. Quitting smoking can significantly reduce the risk of HTN and other CVDs [61]. Chronic stress can contribute to high blood pressure. Engaging in stress-reducing activities like meditation, deep breathing exercises, or hobbies can help manage stress levels [61].

In addition, consuming too much sodium can increase blood pressure. It is recommended to limit sodium intake to less than 2,300 mg per day (or even lower for certain individuals) [61]. If prescribed medication for HTN, it is essential to take it as directed by a healthcare professional and attend regular check-ups [61,62]. Regular visits with a healthcare professional can help monitor blood pressure levels and identify any potential risk factors for HTN [61,62].

Gyamfi et al. discuss the health benefits of the task-shifting strategy for HTN control in Ghana [63]. The study found that nurses who received training in the management and control of HTN showed a marked improvement in their knowledge and practice related to HTN detection and treatment [63]. Specifically, the nurses demonstrated increased proficiency in HTN management, including accurate measurement of blood pressure, initiation of treatment, monitoring of adverse drug reactions, and identification of complicated cases requiring specialist referral [63]. The nurses also reported positive outcomes, such as improved interpersonal skills and patient education [63]. These findings suggest that if all nurses receive even brief training in HTN management, significant public health benefits can be achieved in low-income countries like Ghana.

A study by Zhang et al. mentions the cost-effectiveness of intensive HTN control in China as a preventive strategy for CVD [64]. The study specifically focuses on the cost-effectiveness of folic acid therapy for the primary prevention of stroke in hypertensive patients [64]. It refers to the China Stroke Primary Prevention Trial (CSPPT), which demonstrated that treatment with enalapril-folic acid reduced the risk of primary stroke compared to enalapril alone [64].

Prevention Strategies for CAD

Preventive strategies highlighted by van Bakel et al. include cardiac rehabilitation and physical activity, lifestyle modifications, sedentary behavior intervention, and the use of wearable technology for heart rate and activity tracking [65]. Cardiac rehabilitation and physical activity have been shown to have positive impacts on CAD prevention. They can improve cardiovascular risk factor control, reduce sedentary behavior, increase physical activity levels, and improve the overall quality of life for patients with CAD [65]. Lifestyle modifications, such as a healthy diet, smoking cessation, and weight management, are also important preventive strategies. These lifestyle changes can help control cardiovascular risk factors and reduce the risk of CAD [65].

Elgendy et al. discuss two preventive strategies against CAD: a beta-blocker-based regimen and a calcium antagonist-based strategy [66]. These strategies were used to lower blood pressure and achieve excellent blood pressure control in hypertensive patients with CAD disease [66]. The study found that both strategies were associated with similar long-term benefits in terms of all-cause mortality [66]. There was no significant difference in long-term all-cause mortality between the two treatment strategies [66].

A study by Merino et al. discusses two preventive strategies against CAD: lifestyle intervention and metformin treatment [67]. This strategy involves adopting healthy lifestyle behaviors such as improving diet and increasing physical activity [68]. The study found that participants who underwent lifestyle intervention had greater improvements in various cardiometabolic risk factors (CRFs) compared to those in the placebo group [67]. These improvements included glycemia, anthropometric measures, LDL-C, HDL-C, tissue plasminogen activator (tPA), and CRP [67]. In the study, participants who received metformin treatment also showed significant improvements in glycemia, anthropometric measures, LDL-C, HDL-C, tPA, and CRP compared to the placebo group [67].

Making lifestyle modifications such as regular exercise, a healthy diet, weight management, and smoking cessation can have a significant impact on reducing the risk of CAD [68]. These interventions can provide comprehensive education, promote risk reduction, and improve patient outcomes. The use of health IT systems, including tele-based and web-based interventions, has emerged as an effective alternative model for improving secondary prevention of CAD [68]. Health IT interventions can support risk reduction, patient safety, and lifestyle outcomes. Nurse-led telehealth interventions have been shown to be more efficient than traditional education interventions in improving CAD risk reduction [68].

Therapeutic interventions for CVD prevention

Statins

Dyslipidemia, particularly elevated LDL-C levels, is strongly associated with CVD, especially CAD [69-71]. Large-scale clinical trials in the 1990s onwards consistently show statin therapy's cardiovascular benefits in reducing lipid levels. The Cholesterol Treatment Trialists’ (CTT) meta-analysis in 2005 demonstrated a 21% decrease in major cardiovascular events per 1 mmol reduction in LDL-C, regardless of baseline levels [72]. Subsequent analyses in 2010 reaffirmed these findings and suggested that more intensive statin regimens lead to greater reductions in cardiovascular events [73]. The 2019 ESC/European Atherosclerosis Society (EAS) guidelines advocate aggressive LDL-C reduction for high-risk patients, with goals ranging from <1.4 mmol/L for very high-risk individuals to <3.0 mmol/L for low-risk individuals [74].

Different types and doses of statins offer varying reductions in LDL-C levels, with high-intensity regimens achieving around a 50% reduction and medium-intensity therapy reaching 30-50% [75]. Potent statins like rosuvastatin, pitavastatin, and atorvastatin effectively lower triglyceride levels by 10-20% [76]. Ford et al. demonstrated an 18% reduction in all-cause mortality over more than 20 years in non-diabetic patients with a 10-year ASCVD risk below 7.5% [77]. Statins show efficacy in preventing ASCVD in older adults, but their effectiveness in heart failure and hemodialysis patients is uncertain [63]. Additionally, it has been proposed that the protective impact of statins against ASCVD might extend beyond cholesterol reduction. In the Jupiter trial, rosuvastatin was found to decrease cardiovascular events in individuals with normal LDL-C levels but elevated CRP levels [78]. Beyond cholesterol reduction, statins have been proposed to prevent ASCVD through various mechanisms, including anti-inflammatory, antioxidant, endothelial function-improving, plaque-stabilizing, and platelet aggregation-inhibiting effects [79-81]. The benefits of statin therapy outweigh the risks for patients at high cardiovascular risk, with minimal risks of severe muscle damage, hepatotoxicity, and newly diagnosed DM [82]. Statins significantly reduce the risk of stroke and other adverse cardiovascular events, but there is no convincing evidence linking them to various health issues.

Glucagon-Like Peptide-1 Receptor Agonists

Type 2 DM patients face an increased CVD risk compared to nondiabetic patients, which had already been apparent in the Farmingham and Merit trials [83,84]. Furthermore, CVD in diabetic patients is three times more likely to result in a fatal outcome compared to the general population. GLP-1 exhibits cardiovascular benefits, including effects on blood pressure regulation, vascular endothelial function, atherosclerosis progression, inflammation, myocardial ischemia, and heart failure, which will be discussed in detail later on [85].

In patients with type 2 DM, dyslipidemia is a common and significant comorbidity due to insulin resistance and metabolic dysfunction. This dyslipidemia, termed atherogenic dyslipidemia, is characterized by reduced levels of HDL-C and elevated levels of LDL-C, total cholesterol, and triglycerides. The interplay between dyslipidemia and poor glycemic control is crucial to the progression of atherosclerosis [86]. The Quebec Cardiovascular Study indicates that individuals with DM, high LDL-C, and high apolipoprotein B are at a significantly increased risk, up to 20-fold, of experiencing cardiovascular events [87].

Semaglutide showed a decrease in cardiovascular events and acute cerebral events among patients with an average age of 65 years and an average HbA1c level of 8.7%. However, it exhibited comparable rates of cardiovascular mortality [88]. Both exenatide and liraglutide have been shown to lower total cholesterol and triglyceride values to different degrees [86]. A meta-analysis of the LEAD trials showed that liraglutide reduced total cholesterol, LDL-C, and triglycerides when compared to the standard treatment [89]. The DURATION trials showed similar results with exenatide [86].

Another major contributor to the development of both type 2 DM and CVD is obesity. Patients who experience moderate weight loss have been shown to have better glycemic control and a greater number of medications for controlling cardiovascular risk factors. The AMIGO and DURATION-2 trials have shown weight-loss effects of exenatide [90-92]. Moreover, liraglutide in the LEAD and SCALE trials showed dose-dependent and high-dose regimen weight loss, respectively [93-95]. Similar results were reported for dulaglutide and albiglutide in the AWARD and HARMONY studies, respectively [96-98].

Sodium-Glucose Cotransporter 2 Inhibitors

Several clinical trials have showcased the remarkable cardiovascular benefits of sodium-glucose cotransporter 2 inhibitors (SGLT2i) in both DM and non-DM patients suffering from heart failure. These positive outcomes have spurred various theories aimed at explaining their mechanisms. These theories encompass a broad spectrum of potential advantages, including but not limited to mitigating inflammation and oxidative stress, reducing blood pressure, enhancing weight loss, promoting diuresis and natriuresis, optimizing glycemic control, preventing cardiac remodeling, suppressing the sympathetic nervous system, and improving vascular function while increasing circulating provascular progenitor cells [99-103]. Inflammation significantly contributes to the severity of heart failure, with proinflammatory biomarkers often elevated in affected patients and correlating with disease severity [104,105]. Empagliflozin, canagliflozin, and dapagliflozin have demonstrated the ability to mitigate or improve the inflammatory profile in diabetic patients [106-108]. 

Various trials and meta-analyses have underscored the cardiovascular benefits of SGLT2i in patients with DM and CVD. In the EMPA-REG trial, empagliflozin showed significant reductions in cardiovascular death, hospitalizations for heart failure, and overall mortality among heart failure patients [109]. Similarly, for canagliflozin, the CANVAS trial demonstrated a lower risk of cardiovascular death in DM patients with cardiovascular disease [110,111]. The DECLARE-TIMI 58 (Dapagliflozin Effect on Cardiovascular Events) trial indicated reductions in cardiovascular deaths and heart failure hospitalizations in diabetic patients on dapagliflozin, irrespective of their ASCVD status [112]. The DAPA-HF (Dapagliflozin and Prevention of Adverse Outcomes in Heart Failure) trial results suggested that dapagliflozin reduced cardiovascular mortality and heart failure hospitalizations in all patients, regardless of DM diagnosis [113]. The CREDENCE (Canagliflozin and Renal Events in Diabetes With Established Nephropathy Clinical Evaluation) trial further confirmed the cardiovascular benefits of SGLT2i in diabetic patients with chronic kidney disease [114]. Meta-analyses have consistently shown reductions in major adverse cardiovascular events, cardiovascular death, and all-cause mortality with SGLT2i treatment [115-117]. These findings indicate that SGLT2i offers cardiovascular protection beyond its glucose-lowering effects, although their precise mechanism of action requires further investigation [118].

Proprotein Convertase Subtilisin/Kexin Type 9 Inhibitors

Patients with dyslipidemia, especially those with high LDL-C, carry a significant risk factor for ASCVD [73,119]. To control LDL-C levels and avoid future CVD in those patients, statins have always been considered the first-line treatment [120]. However, there has always been an urgent need for new agents to further control unmanageable and unresponsive LDL-C levels in high-risk patients. Proprotein convertase subtilisin/kexin type 9 (PCSK9) inhibitors, which were approved within the last decade, have been included in the most recent guidelines to be combined with ezetimibe and statin drugs to lower cardiovascular risk in these individuals [120]. Normally, PCSK9 promotes the breakdown of LDL-C receptors, causing decreased clearance of LDL-C from circulation. PCSK9 inhibitors function by modulating LDL-C receptor expression on hepatocytes’ surfaces; thus, they may decrease LDL-C and, subsequently, significant CVD [121]. The primary indication for PCSK9 is secondary prevention among patients with a very high risk of ASCVD events [122]. It has been shown that PCSK9 inhibitors are potent agents for the lowering of LDL-C. Furthermore, when compared to statins, PCSK9 inhibitors resulted in a greater decrease in LDL-C [123,124]. As a monotherapy, PCSK9 inhibitors have been shown to reduce LDL-C by 47-57% [125,126]. PCSK9 inhibitors, evolocumab and alirocumab, have been shown to reduce LDL-C from baseline, ranging from 36% to 62% at 24 weeks when added to high intensity or maximally tolerated statin therapy [127-129]. Additionally, when combined with statins, PCSK9 inhibitors have been shown to improve cardiovascular outcomes. The ODYSSEY OUTCOMES trial showed that adding alirocumab to maximally tolerated statin therapy reduces the risk of CVD [130]. Comparatively, in the FOURIER trial, evolocumab was shown to lower the risk of CVD when combined with statin medication [131].

When choosing appropriate therapy, the 2019 ESC/EAC guidelines recommend a stepwise approach, starting with a high-intensity statin (maximally tolerated) with the addition of ezetimibe, followed by PCSK9 inhibitor therapy in order to reach the LDL-C goals. However, it is also possible to directly add PCSK9 inhibitors to statin therapy [74]. In comparison with the average LDL-C reduction from combining a high-intensity statin and ezetimibe, which is around 65%, adding a PCSK9 inhibitor to a high-intensity statin can reduce LDL-C by an average of 75% [74]. The 2018 ACC/AHA Multi-society Guideline on the Management of Blood Cholesterol recommends the use of PCSK9 inhibitors for secondary prevention patients at very high risk of ASCVD [132]. In addition, it also recommends the initiation of PCSK9 inhibitors on top of high-intensity or maximally tolerated statin therapy and ezetimibe if LDL-C remains ≥70 mg/dL or non-HDL-C remains ≥100 mg/dL [133].

## Conclusions

In summary, proactive measures are crucial for preserving cardiovascular health amid the interconnected risks of HTN, DM, dyslipidemia, and CAD. Lifestyle adjustments, including a heart-healthy diet, exercise, weight control, stress management, and tobacco/alcohol cessation, play a fundamental role in preventing HTN and CAD. Regular monitoring of blood pressure, cholesterol, and overall cardiovascular health allows for early detection and targeted management of risk factors. Embracing these preventive measures, alongside medical guidance, substantially reduces the risks of HTN, CAD, and associated complications, promoting enhanced heart health and overall wellness. Future directions in prevention research involve exploring innovative technologies, personalized prevention strategies based on genetics, improved remote monitoring, refining risk prediction models, addressing healthcare disparities, and implementing cost-effective preventive measures globally.
